# Highly Stable Gully-Network Co_3_O_4_ Nanowire Arrays as Battery-Type Electrode for Outstanding Supercapacitor Performance

**DOI:** 10.3389/fchem.2018.00636

**Published:** 2018-12-21

**Authors:** Chunli Guo, Minshuai Yin, Chun Wu, Jie Li, Changhui Sun, Chuankun Jia, Taotao Li, Lifeng Hou, Yinghui Wei

**Affiliations:** ^1^College of Materials Science and Engineering, Taiyuan University of Technology, Taiyuan, China; ^2^College of Materials Science and Engineering, Changsha University of Science & Technology, Changsha, China; ^3^School of Chemistry and Chemical Engineering, Qilu Normal University, Jinan, China; ^4^Key Laboratory of Advanced Energy Materials Chemistry (Ministry of Education), Nankai University, Tianjin, China; ^5^College of Materials Science and Engineering, Taiyuan University of Science and Technology, Taiyuan, China

**Keywords:** Co_3_O_4_ nanowire arrays, 3D gully-network structure, hybrid-supercapacitor, stable cycle performance, battery-type electrode

## Abstract

3D transition metal oxides, especially constructed from the interconnected nanowires directly grown on conductive current collectors, are considered to be the most promising electrode material candidates for advanced supercapacitors because 3D network could simultaneously enhance the mechanical and electrochemical performance. The work about design, fabrication, and characterization of 3D gully-network Co_3_O_4_ nanowire arrays directly grown on Ni foam using a facile hydrothermal procedure followed by calcination treatment will be introduced. When evaluated as a binder-free battery-type electrode for supercapacitor, a high specific capacity of 582.8 C g^−1^ at a current density of 1 A g^−1^, a desirable rate capability with capacity retention about 84.8% at 20 A g^−1^, and an outstanding cycle performance of 93.1% capacity retention after 25,000 cycles can be achieved. More remarkably, an energy density of 33.8 W h kg^−1^ at a power density of 224 W kg^−1^ and wonderful cycling stability with 74% capacity retention after 10,000 cycles can be delivered based on the hybrid-supercapacitor with the as-prepared Co_3_O_4_ nanowire arrays as a positive electrode and active carbon as negative electrode. All the unexceptionable supercapacitive behaviors illustrates that our unique 3D gully-network structure Co_3_O_4_ nanowire arrays hold a great promise for constructing high-performance energy storage devices.

## Introduction

With the rapid depletion of fossil fuels and deterioration of the environment, an urgent demand has emerged for the development of sustainable and clean energy storage devices in terms of high power density, energy density, and good safety performance (Wang et al., 2012b; Ren et al., 2015; Zhang et al., 2018; Zeng et al., 2019). Because of the admirable supercapacitive behaviors, ecological features, and safety characteristics, supercapacitors have been extensively considered as the major devices for energy storage adhibition thanks to the great potential as power sources for various applications, from electric vehicles to smart grids (Raj et al., 2015; Yan et al., 2017; Wu et al., 2018). As documented in the previous investigations that the supercapacitive behaviors of these devices heavily rely on the properties of electrode materials (Chang et al., 2016; Cui et al., 2019). Therefore, the vital point to acquire wonderful behaviors of these devices is to hunt for proper materials and suitably design the electrode structure (Xu et al., 2017).

Because of the suitability for large-scale fabrication, high specific capacity, and rich redox reactions involving different ions, the transition metal oxides have been intensively studied as the most potential electrode materials in supercapacitors, which are divided into a capacitive electrode materials (MnO_2_ and RuO_2_) having a quasi-rectangular cyclic voltammetry (CV) curve and a battery-type electrode materials (cobalt and nickel based compounds) having a strong separated redox peaks by the shape of the CV curve (Huang et al., 2014; Shao et al., 2015; Wang et al., 2015; Dai et al., 2017, 2018; Jiang et al., 2017; Wu et al., 2019). In particular, cobalt oxide, as a typical battery-type electrode material, has attracted a great deal of attention due to its low cost, natural abundance, high surface area structural characteristics, and high theoretical capacity (Yuan et al., 2012; Kuang et al., 2015; Jiang et al., 2016). Wang et al. successfully prepared Co_3_O_4_ nanostructures with a specific capacity of 354.6 C g^−1^ at 0.5 A g^−1^, and the cycle performance in particular is about 97% after 2,000 cycles with a current density of 1 A g^−1^ (Wang et al., 2016). Well-crystalline porous cobalt oxide (Co_3_O_4_) nanorods (NRs) synthesized by hydrothermal method by Shin's group (Jang et al., 2017). A specific capacity about 316.4 C g^−1^ at 10 mV s^−1^ can be achieved based on porous Co_3_O_4_ NRs-300°C electrode. In addition, about 76% capacity retention of the porous Co_3_O_4_ NRs electrode can be maintained after 5,000 cycles. 3D-nanonet hollow structured Co_3_O_4_ electrode for battery-type supercapitor, prepared by a facile, low cost, and eco-friendly route under ambient temperature and pressure, can exhibit 410, 377.28, 346.68, and 328 C g^−1^ under various scan rates about 5, 10, 20, and 30 mV s^−1^, respectively (Wang et al., 2014). The as-prepared electrode also reveals good stability and keeps 90.2% of its initial capacity after 1,000 cycles at 5 A g^−1^. However, there is a big distance in the obtained Co_3_O_4_ electrode reaching to the theoretical specific capacity and a better cycling stability owing to the intrinsic highly resistive nature or the agglomeration of the Co_3_O_4_ nanomaterials during the electrode fabrication process (Rajeshkhanna et al., 2016; Wang et al., 2016; Kong et al., 2017).

One effective way proved to avoid the disadvantages caused by the binder addition and enhance the electrochemical performances of the Co_3_O_4_ electrodes is to grow the active electrode materials directly on conductive substrates such as Ni foam (Mao et al., 2016). Zhang et al. confirmed that Co_3_O_4_ nanowire array grown directly on Ni foam, fabricated by a simple hydrothermal method together with a post-annealing process, exhibited a specific capacity of 580 C g^−1^ at 1 A g^−1^ and a cycle performance of 90.6% after 5,000 cycles at 8 A g^−1^ (Zhang et al., 2012). Gao et al. also reported that 3D star-shaped Co_3_O_4_ nanowire growing on Ni foam prepared by a simple hydrothermal method displayed the specific capacity of 700 C g^−1^ at 1 A g^−1^. However, the specific capacity retention rate is 92.26% after only 1,000 cycles at a current density of 12.5 A g^−1^ (Gao et al., 2016). Qing et al. investigated that Co_3_O_4_ nanoflower structure on Ni foam showed a high specific capacity of 514.8 C g^−1^ at 1 A g^−1^, but its cycle performance was only 78.2% at 3 A g^−1^ after 1,000 cycles (Qing et al., 2011). As a result, the integrated electrodes exhibited the improvement of the specific capacity, but their poor cycling stability was far from solved, which partly resulted from the large volume changes of Co_3_O_4_ electrodes during the repeated cycle process (Yao et al., 2011), leading to structural instability, active material shedding off, and the capacity decay.

To address the above issues and achieve the excellent supercapacitive electrode materials, herein, we report our findings about the design, fabrication, and characterization of 3D gully-network structure Co_3_O_4_ nanowire arrays directly grown on Ni foam through a facile and scalable hydrothermal procedure followed by annealing treatment. It has been demonstrated that the direct growth of 3D gully-network structure on Ni foam could ensure that every nanowire possesses good mechanical adhesion and participates in electrochemical reactions, so a stable 3D mechanical network benefits most to its excellent cycling ability. More importantly, good electrical contact with the current collector, which would result in low contact resistance and wonderful supercapacitive behaviors in terms of high specific capacity, admirable rate capability as well as outstanding cycle stability.

## Experimental

### Preparation of 3D Gully-Network Co_3_O_4_ Nanowires on Ni Foam

All the purchased chemicals were analytical grade and used without further purification. The Ni foam was cleaned ultrasonically for 15 min in 3M HCl solution, absolute ethanol, and deionized water to remove the surface oxide layer and was dried in a vacuum oven for 2 h. 0.4851 g Co(NO_3_)_2_ and 0.0912 g urea were dissolved in 40 mL deionized water. Next, 0.0834 g cetyltrimethylammonium bromide (CTAB) and 0.0133 g sodium dodecyl benzene sulfonate (SDBS) were added to the above mixed solution and were stirred magnetically for 15 min, respectively. Then, both the mixed solution and the ready-prepared Ni foam were transferred into a 60 mL Teflon-lined stainless steel autoclave, sealed, and placed in an electric oven at 140°C for 4 h, and then allowed to cool to room temperature naturally. The precursors and precipitates were collected and washed three times with absolute ethanol and deionized water, respectively, then dried at 60°C overnight. Finally, the prepared precursor was annealed at 300°C in air for 2 h with a heating rate of 1°C min^−1^ to obtain 3D gully-network Co_3_O_4_ nanowires (NWAs) on Ni foam. The mass loading of Co_3_O_4_ NWAs on Ni foam was about 2.1 mg cm^−2^.

### Preparation of the Hybrid-Supercapacitor

The hybrid-supercapacitor was assembled by the Co_3_O_4_ NWAs on Ni foam as the positive electrode, the activated carbon (AC) as the negative electrode, polypropylene separator as the separator, 6 M KOH as the electrolyte. Specifically, the negative electrode was prepared as follows: First, AC and polytetrafluoroethylene (PTFE) (mass fraction: 5%) were mixed in a ratio of 9:1 and ultrasonically diluted with anhydrous ethanol. The Ni foam (3 × 3 cm) was soaked in the mixed solution for 3 min. Finally, the AC was uniformly distributed on Ni foam after drying in air at 60°C for 6 h, and the mass loading of AC electrode was 2.4 mg cm^−2^. Positive and negative electrodes in hybrid-supercapacitor must meet charge balance (q^+^ = q^−^). The charge contained in each electrode is calculated using the following equation [Equation (1)]:

(1) $$\begin{array}{l} \text{q }=\;{\text{Q}}_{\text{ua}}\;\times \;{\text{m}}_{\text{ua}}\;\times \text{ S} \end{array}$$

Where *Q*_ua_ (C g^−1^) is the specific capacity per unit area of the electrode, *m*_ua_ (g) is the mass per area (cm^2^) of the active material and S (cm^2^) is the area of the electrode.

In order to achieve q^+^ = q^−^, the area ratio satisfies equation (2):

(2) $$\begin{array}{l} \frac{{\text{S}}_{+}}{{\text{S}}_{-}}=\frac{{\text{Q}}_{\text{ua}-}\;\times {\text{ m}}_{\text{ua}-}}{{\text{Q}}_{\text{ua}+}\;\times {\text{ m}}_{\text{ua}+}} \end{array}$$

According to the specific capacity of both the positive and the negative electrode, the best positive and negative electrode area ratio was found to be S^−^/S^+^ = 3. Finally assembled into hybrid-supercapacitor.

### Materials Characterization

Scanning electron microscope (SEM) analyses were performed using a SU8010 SEM, operated at 10 kV. Transmission electron microscope (TEM) images and selected area electron diffraction (SAED) patterns were collected on JEOL-2100F at 200 kV. X-ray diffraction (XRD) using Cu-K*a* radiation (K*a* = 1.5418 Å). Raman scattering spectra were taken on a SPEX-1403 Raman spectrometer (Ar ion laser, 514.5 nm). X-ray photoelectron spectra (XPS) were recorded on an Axis Ultra, Kratos (UK) spectrometer, using a standard Al Kα X-ray source (150 W) and an analyzer pass energy of 30 eV. Samples were mounted using double-sided adhesive tape and binding energies are referenced to the C (1 s) binding energy of adventitious carbon contamination taken to be 284.8 eV.

### Electrochemical Measurements

The CV, galvanostatic charge/discharge (GCD), and electrochemical impedance spectroscopy (EIS) techniques were used to evaluate the electrochemical performance of Co_3_O_4_ NWAs and hybrid-supercapacitor on a CHI660E electrochemical workstation at room temperature. Co_3_O_4_ NWAs were tested in a three-electrode system where Hg/HgO electrode is the reference electrode, Pt electrode (3 × 3 cm) is the counter electrode and the preparation electrode material is the working electrode. However, hybrid-supercapacitors were tested in a two-electrode system with Co_3_O_4_ NWAs as the positive electrode and AC as the negative electrode. All electrochemical tests were performed in 6M KOH. We calculated the specific capacity (Q_sc_) (C g^−1^), energy density (E) (Wh Kg^−1^), and power density (P) (W Kg^−1^) based on the discharge time in the GCD curve and calculate it according to Equations (3–5).

(3) $$\begin{array}{l} {\text{Q}}_{\text{sc}}=\frac{\text{It}}{\text{m}} \end{array}$$

(4) $$\begin{array}{l} \text{E}=\frac{1}{2}{\text{Q}}_{\text{sc}}\text{V} \end{array}$$

(5) $$\begin{array}{l} \text{P}=\frac{\text{E}}{\text{t}} \end{array}$$

Where I (A) is the discharge current, t (s) is the discharge time, V (V) is the voltage window, and m (g) is the mass of the electrode material.

## Result and Discussion

**Figure 1:**
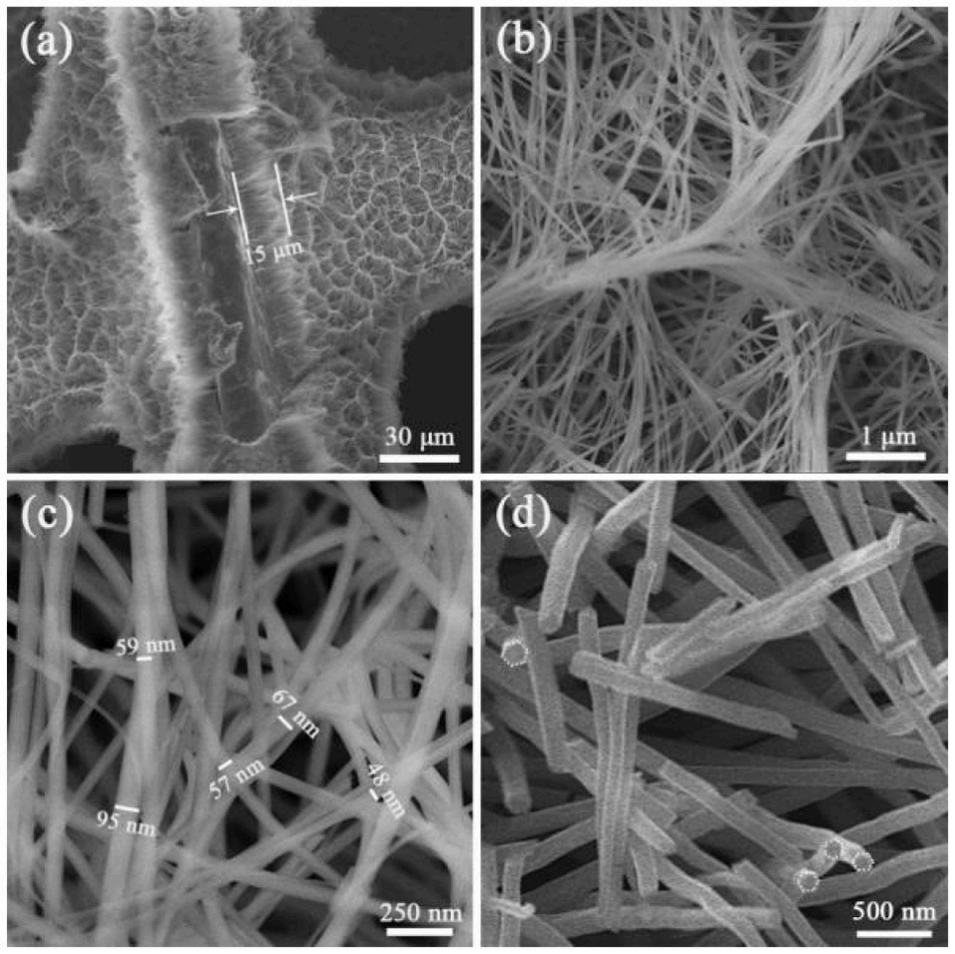
**(a–c)** SEM images of precursor nanowire arrays on Ni foam at different magnifications, **(d)** the cross-sectional SEM image of the precursor nanowires.

Figure 1 was the SEM images of a precursor nanowire array on Ni foam. Figure 1a presents that the precursor nanowire array uniformly grows on the Ni foam surface. According to the missing part, the thickness of the nanowire array is about 15 μm. It is worth noting that the top of the nanowire is bent instead of perpendicular to the surface of the Ni foam. It is further observed (Figure 1a) that the tops of the nanowires were clustered together to form a ridge-like structure, and the connecting ridges eventually formed an irregular gully-network structure. Figure 1b confirms the top of each precursor nanowire gathering and fixing on the ridge to form a stable structure. Figure 1c shows that the precursor nanowires cross each other with a diameter of 50–100 nm, further improving the structure stability of the precursor nanowire array. Figure 1d displays the cross-sectional of the precursor nanowires. The cross-sectional presents a hexagon. It is possible to add anionic and cationic surfactants in a certain proportion during the preparation to make the solution more polar, which limits the lateral growth of nanowires (Stellner et al., 1986; Wu et al., 2013; Zhang et al., 2016). The precursor is converted into Co_3_O_4_ by the heat treatment. During the heat treatment, the precursor thermally decomposes to produce H_2_O and CO_2_ [reaction (6)], which is derived from the slow hydrolysis of urea [reaction (7)–(9)]. It has been reported that the slow hydrolysis of urea facilitates the growth of nanowires and has better crystallinity along the longitudinal axis. Synthesized ultra-long nanowires benefit from the addition of urea (Wang et al., 2012a). The hydrolyzed ${\text{CO}}_{\text{3}}^{\text{2-}}$ and OH^−^ combine with Co^2+^ to form the precipitate Co(CO_3_)_0.5_(OH)·0.11H_2_O (reaction (10): (Fan et al., 2017).

(6) $$\begin{array}{l} 3\text{Co}{\left(\text{C}{\text{O}}_{3}\right)}_{0.5}\left(\text{OH}\right)\cdot 0.11{\text{H}}_{2}\text{O}\overset{\Delta }{\to }\text{C}{\text{o}}_{3}{\text{O}}_{4}\\ +1.83{\text{H}}_{2}\text{O↑}+1.5\text{C}{\text{O}}_{2}\text{↑} \end{array}$$

(7) $$\begin{array}{l} {\text{H}}_{2}\text{NCON}{\text{H}}_{2}+{\text{H}}_{2}\text{O }\to \;2\text{N}{\text{H}}_{3}\text{↑}+\text{C}{\text{O}}_{2}\text{↑} \end{array}$$

(8) $$\begin{array}{l} \text{C}{\text{O}}_{2}+{\text{H}}_{2}\text{O }\to \text{ C}{\text{O}}_{3}^{2-}+2{\text{H}}^{+} \end{array}$$

(9) $$\begin{array}{l} \text{N}{\text{H}}_{3}+{\text{H}}_{2}\text{O }\to \text{ N}{\text{H}}_{4}^{+}+\text{O}{\text{H}}^{-}\\ \text{C}{\text{o}}^{2+}+0.5\text{C}{\text{O}}_{3}^{2-}+\text{O}{\text{H}}^{-}+0.11{\text{H}}_{2}\text{O }\to \text{ Co}{\left(\text{C}{\text{O}}_{3}\right)}_{0.5} \end{array}$$

(10) $$\begin{array}{l} \left(\text{OH}\right)\cdot 0.11{\text{H}}_{2}\text{O↓} \end{array}$$

**Figure 2:**
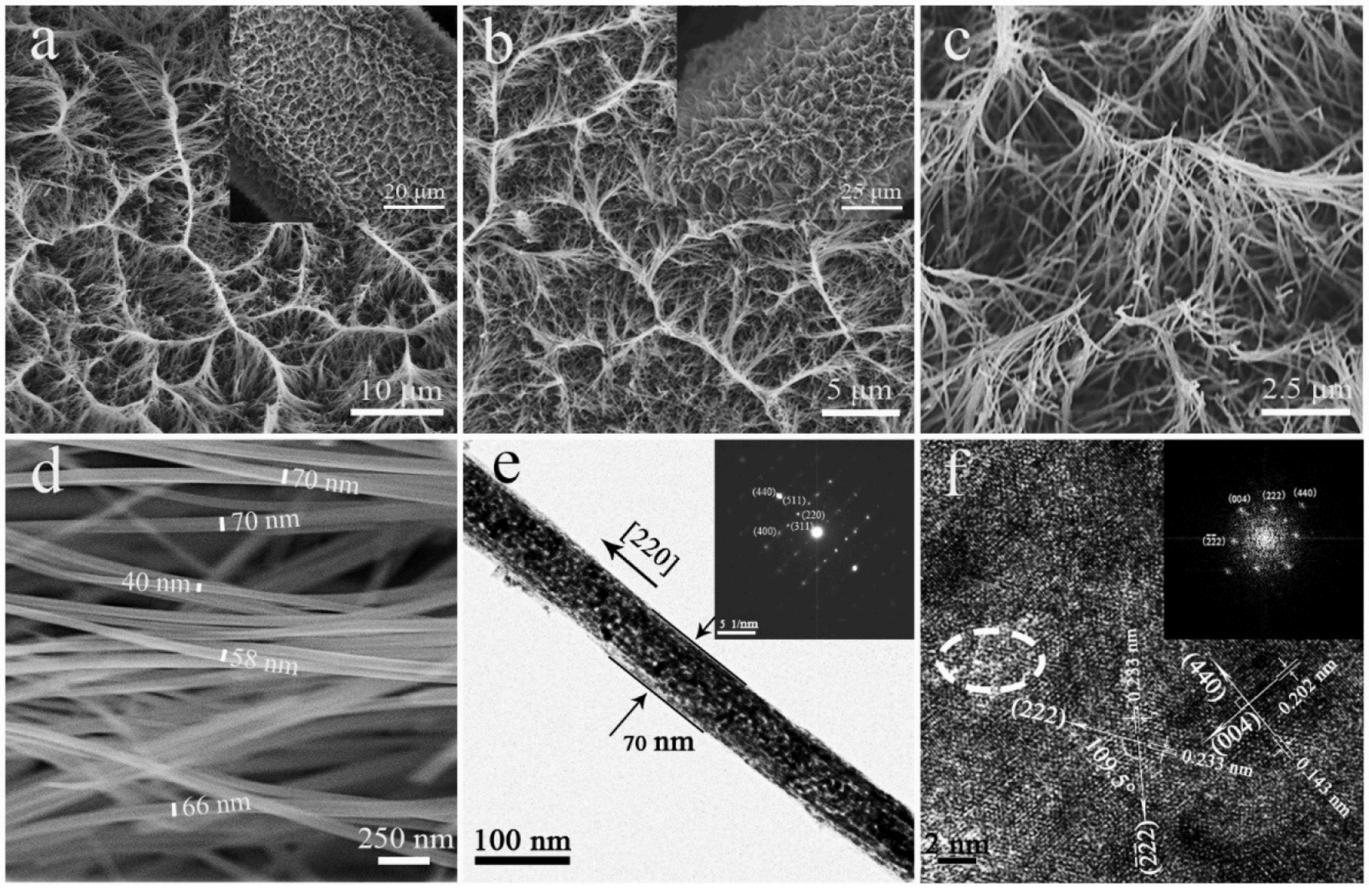
**(a)** SEM images of precursor nanowire arrays on Ni foam, **(b–d)** SEM images of Co_3_O_4_ NWAs on Ni foam at different magnifications, **(e)** TEM images (inset: SAED pattern) of Co_3_O_4_ nanowires, **(f)** HRTEM image of Co_3_O_4_ nanowires and corresponding FFT images (inset).

The SEM images of Co_3_O_4_ NWAs on Ni foam obtained after annealing are shown in Figures 2b–f. Comparing Figures 2a,b, it can be seen that the gully-network structure does not change significantly before and after annealing. Because the gully-network structure makes the nanowires cross each other so that the nanowires can be fixed. Therefore, it has a good spatial buffer to adapt to the volume change in the annealing process. Further magnification of Figure 2c shows the sides of the gully-network structure, where the nanowires gather along the gully orderly and form a hemp rope structure at the top, so that the nanowire array forms a tight whole. As shown in Figure 2d, the Co_3_O_4_ nanowires diameter is 40–70 nm, which is 10–30 nm smaller than the precursor nanowire, owing to the loss of CO_2_ and H_2_O during annealing treatment (Guan et al., 2017).

Co_3_O_4_ nanowires further characterized by TEM equipped with HRTEM and SAED as shown in Figures 2e,f. The surface of the Co_3_O_4_ nanowires (Figure 2e) is uneven and highly porous, providing more specific surface area and active sites. The SAED plot (inset in Figure 2e) displays the single crystal diffraction spots of Co_3_O_4_ nanowires, and diffraction spots of (220) (311) (511) (400) and (440) are observed. The long axis direction of the nanowires is perpendicular to the (220) crystal plane and the nanowires grow along the (110) crystallographic axis. It is worth noting that the crystal growth direction is different from that in other articles along the (111) direction (Xia et al., 2012). HRTEM image of the middle part of the Co_3_O_4_ nanowire (Figure 2e) is shown in Figure 2f. The lattice spacing of the vertical lattice fringes is 0.202 and 0.143 nm corresponds to the (004) and (440) crystal planes of Co_3_O_4_ (JCPDS card no.42-1467). In addition, there are two crystal planes with the same lattice spacing of 0.233 nm on the HRTEM image, corresponding to the (222) and ($\overset{-}{2}\overset{-}{2}$2) crystal planes of Co_3_O_4_ with the crystal plane angle of 109.5°. The FTT corresponding to HRTEM is shown in the inset in Figure 2f, which exhibits the (222) and ($\overset{-}{2}\overset{-}{2}$2) crystal planes compared to SAED. Obviously noticed that there is no complete and clear lattice fringes in the dotted circle. The dashed circle may be a surface defect because of the loss of CO_2_ and H_2_O during the annealing treatment, which has high electrochemical activity and improves electrochemical performance.

**Figure 3:**
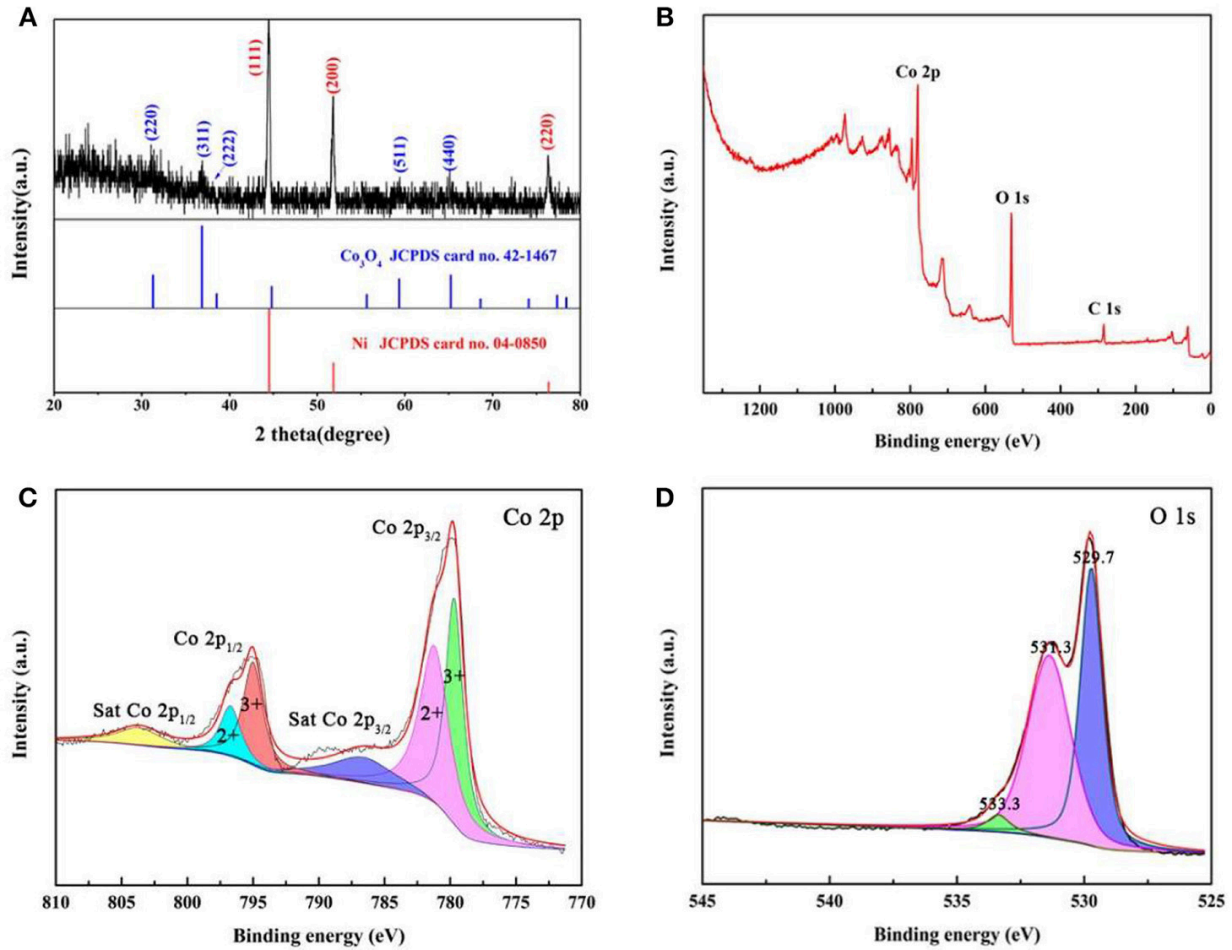
**(A)** XRD patterns of Co_3_O_4_ NWAs on Ni foam, **(B)** XPS spectra of Co_3_O_4_ NWAs, **(C)** XPS survey scan of Co 2p, and **(D)** XPS survey scan of O 1 s.

XRD, Raman spectrum, and XPS are used to analysis the crystal structure of Co_3_O_4_ NWAs. The XRD pattern of the precursor in Figure S1A is consistent with Co(CO_3_)_0.5_(OH)·0.11H_2_O (JCPDS card no. 48-0083) (Xiong et al., 2012). After annealing, the XRD pattern is shown in Figure 3A. Except for the three diffraction peaks of the Ni foam (JCPDS card no. 04-0850) (Yu et al., 2015), the remaining diffraction peaks exactly corresponds to Co_3_O_4_ (JCPDS card no. 42-1467) (Che et al., 2014). Figure S1B indicates the Raman spectra with Raman peaks appearing at 476, 516, 605, 675 cm^−1^ corresponding to E_g_, ${\text{F}}_{2\text{g}}^{1}$, ${\text{F}}_{2\text{g}}^{2}$, and A_1g_ peaks of Co_3_O_4_ (Kim et al., 2011). To study the chemical bonding state of Co_3_O_4_ NWAs, XPS measurement is utilized. Three elements of Co, O, and C in the XPS spectrum (Figure 3B) can be noted and no other elements are found, indicating that there are no impurities in the synthesis of Co_3_O_4_ NWAs. Figure 3C shows the XPS spectrum of Co2p with two sets of peaks, Co 2p_3/2_ (781.2 eV/779.7 eV) and Co 2p_1/2_ (796.7 eV/795.0 eV), which are consistent with Co^2+^/Co^3+^ in Co_3_O_4_ NWAs, respectively (Hu et al., 2016; Zhang et al., 2019). Two satellite peaks of sat.Co 2p_3/2_ (786.6 eV) and sat.Co 2p_1/2_ (803.6 eV) are also observed (Li et al., 2014). The O 1 s spectrum (Figure 3D) consists of 529.7, 531.3, and 533.3 eV. These three peaks represent the O element in Co_3_O_4_, the O element in the water adsorbed on the surface, and the oxygen vacancies formed by gas escape during annealing, respectively (Xiong et al., 2009; Qu et al., 2017). Co_3_O_4_ NWAs is further demonstrated the successful synthesis by XPS.

**Figure 4:**
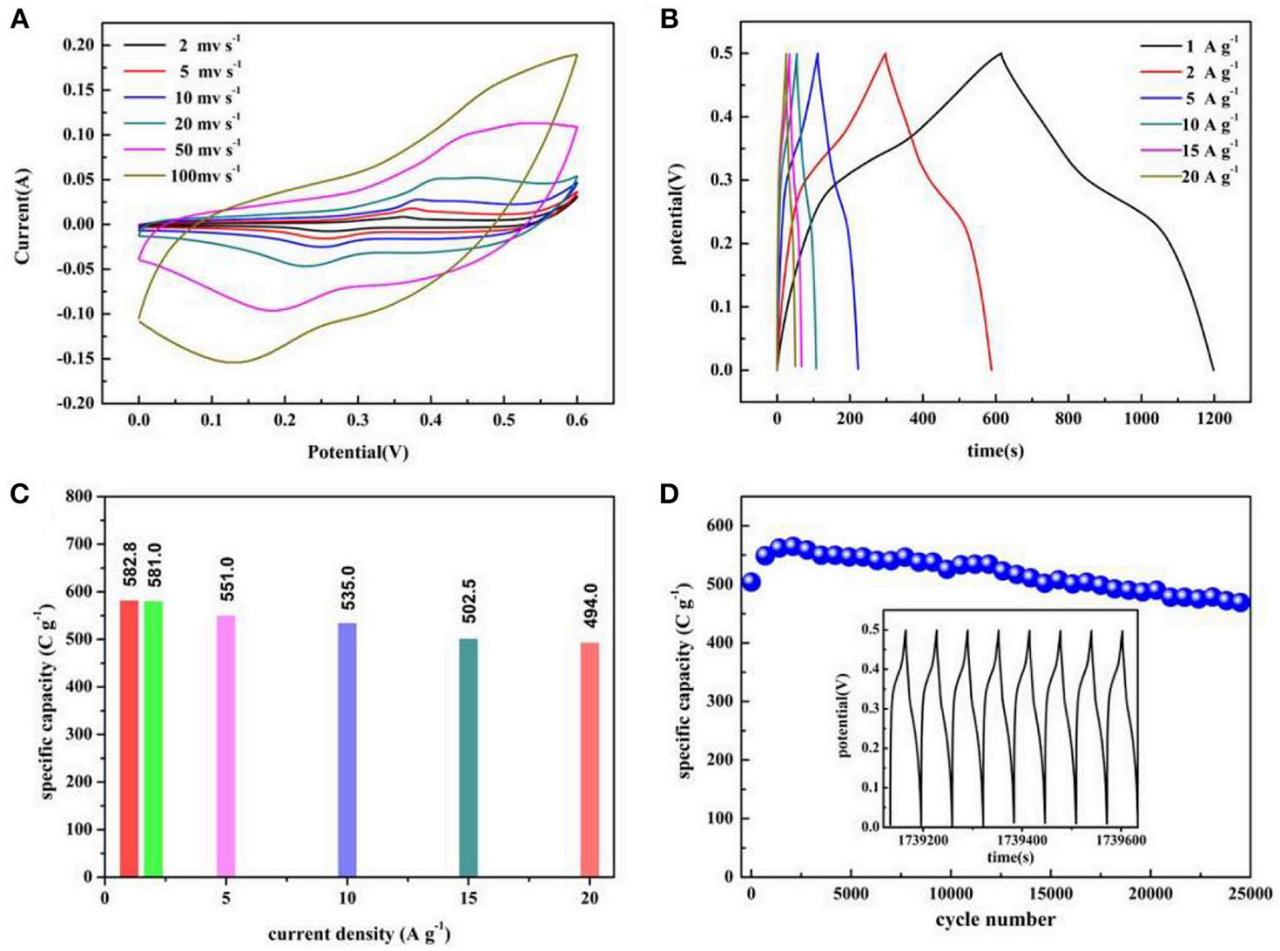
**(A)** CV curves of the Co_3_O_4_ NWAs electrode at various scan rates, **(B)** Galvanostatic charge-discharge curves of the Co_3_O_4_ NWAs electrode at different current densities, **(C)** Specific capacitance of the Co_3_O_4_ NWAs electrodes at different current densities. **(D)** Long-term cycling stability of the Co_3_O_4_ NWAs electrode at 15 A g^−1^(The inset shows the galvanostatic charge-discharge curves of the last 8 cycles).

Electrochemical performance evaluations of Co_3_O_4_ NWAs with gully-network structure are conducted by CV, GCD, and EIS techniques. As shown in Figure 4A, the CV curve of Co_3_O_4_ NWAs on Ni foam obtained in potential range of 0–0.6 V at the scan rate from 2 to 100 mv s^−1^ shows a pair of reversible redox peaks at 0.36 and 0.26 V, displaying a typical faraday behavior (Guan et al., 2017). It is known that the redox reaction occurs between Co_3_O_4_ and OH^−^ in the electrolyte. No OH^−^ in the electrolyte, no redox reaction occurs. To confirm the above conclusion, the electrochemical tests of Co_3_O_4_ NWAs under alkaline and neutral electrolyte conditions are shown in Figure S2. The results from Figure S2a show that the area of the CV curve in the alkaline electrolyte is significantly larger than that in the neutral electrolyte. It indicates that although the Co_3_O_4_ NWAs possesses a large specific surface area, the provided double layer capacitance is very limited, so the faraday behavior is dominant. Meanwhile, the reversible redox reactions occurred are as follows:

(11) $$\begin{array}{l} \text{C}{\text{o}}_{3}{\text{O}}_{4}\;+\text{ O}{\text{H}}^{-}\;+\;{\text{H}}_{2}\text{O }↔\;3\text{CoOOH }+\;{\text{e}}^{-} \end{array}$$

(12) $$\begin{array}{l} \text{CoOOH }+\text{ O}{\text{H}}^{-}\;↔\text{ Co}{\text{O}}_{2}\;+\;{\text{H}}_{2}\text{O }+\;{\text{e}}^{-} \end{array}$$

**Figure 5:**
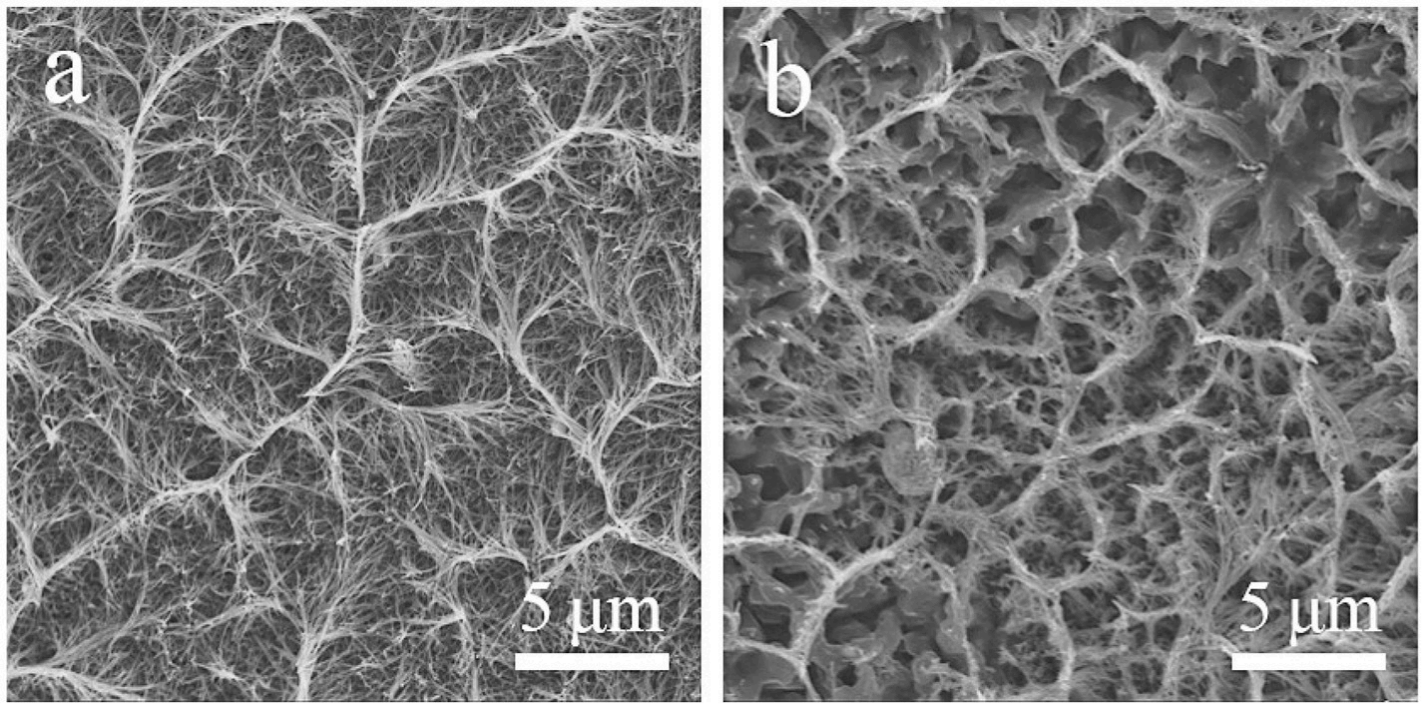
SEM images of Co_3_O_4_ NWAs electrode before **(a)** and after **(b)** 25,000 cycles.

It is noteworthy that the anode and cathode peaks move slightly toward the high and low potentials, respectively, as the scan rate increases. The above phenomenon can be ascribed to the internal ion diffusion resistances enhancing with the increase of the scan rates. However, there is no significant shift in the redox peak, indicating that the electrode exhibits good reaction kinetics (Wang et al., 2012b; Raj et al., 2015). In addition, the nanowire array has a porous structure, which facilitates rapid reversible oxidation-reduction reactions. Figure 4B displays the GCD curves of Co_3_O_4_ NWAs at different current densities under 0–0.5 V, which presents the excellent symmetry of the charge-discharge curves and no significant voltage drop can be observed, indicating the small electrode resistances of the electrodes. Figure 4C shows the specific capacity values at different current densities calculated from the discharge time in Figure 4B. It is clearly observed that a high specific capacity is 582.8 C g^−1^ at a current density of 1 A g^−1^ can be achieved. The specific capacity still maintains 84.8% with the value of 494 C g^−1^ when the current density increases to 20 A g^−1^, which reveals the excellent rate performance of the Co_3_O_4_ NWAs electrode. Furthermore, the long-term cycling stability of the Co_3_O_4_ NWAs electrode at 15 A g^−1^ is presented in Figure 4D. As the cycle progressed, the capacity showed a trend of first increase and then decrease behaviors. The initial increase can be attributed to the activation process of the Co_3_O_4_ NWAs electrode. After that, the slow decay started due to the inactivation of the structure. More remarkably, the capacity still maintains 93.1% after 25,000 cycles, indicating excellent long-term cycling stability. Compared with the related literatures, the 3D gully-network structure Co_3_O_4_ NWAs has the superior electrochemical performances, especially the cyclic stability (Table 1). The inset in Figure 4D shows the final GCD curve during the cycles, which is basically consistent with that before the cycles, demonstrating wonderful reversibility of the Co_3_O_4_ NWAs electrode. The SEM images before and after 25,000 cycles are exhibited in Figure 5, from which the microstructure of the Co_3_O_4_ NWAs remained essentially intact, indicating the outstanding stability of the gully-network structure. With the unique structure, the electrode would possess excellent adaptability to the volume expansion and contraction during the cycle, thus resulting in the brilliant cycling performance.

**Table 1:** Comparison of the specific capacity for Co_3_O_4_ nanomaterials.

**Electrode materials**	**Capacity (C g^**−1**^)**	**Capacity retention**	**References**
Co_3_O_4_ nanowires	278.4	85% after 1,000 cycles	Liu et al., 2015
Co_3_O_4_ hollow nanocubes	159.1	95% after 2,000 cycles	Zhao et al., 2015
Mesoporous Co_3_O_4_/carbon composites	92.4	93.4% after 2,000 cycles	Balasubramanian and Kamaraj, 2015
Co_3_O_4_ nanoflakes	576.8	82% after 5,000 cycles	Kong et al., 2017
Porous Co_3_O_4_ sheets	144	79% after 2,000 cycles	Jing et al., 2015
Co_3_O_4_@NiCo_2_O_4_ core-shell arrays	268.8	97.1% after 3,000 cycles	Gao et al., 2014
Co_3_O_4_/SnO_2_@MnO_2_ core-shell	225	90.7% after 6,000 cycles	Huang et al., 2015
Gully-network Co_3_O_4_ NWAs	582.8	93.1% after 25,000 cycles	This work

**Figure 6:**
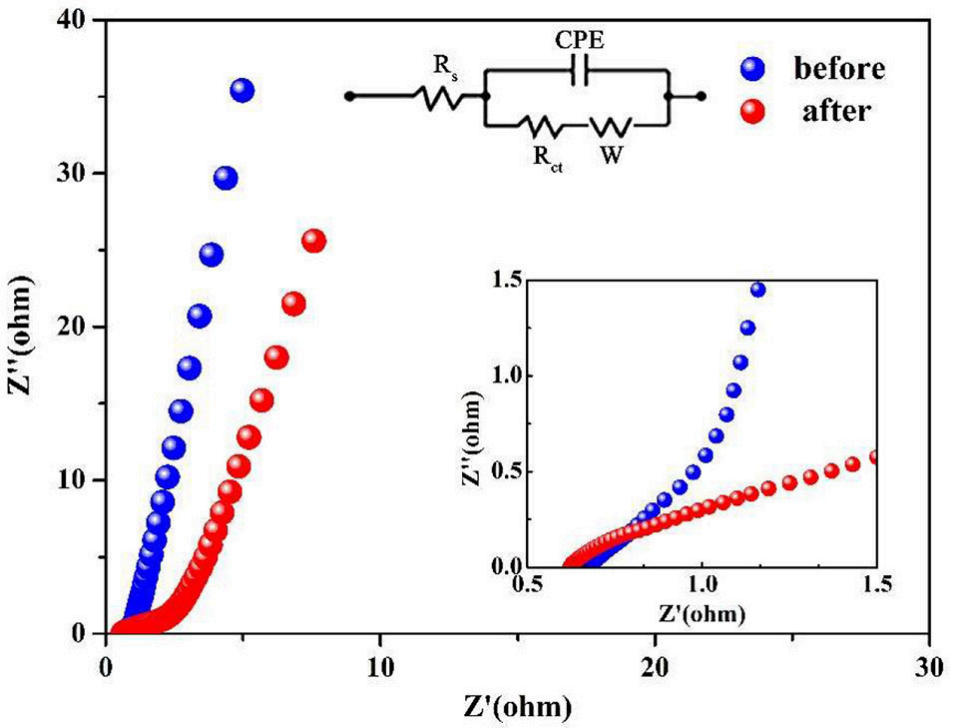
Nyquist plots of Co_3_O_4_ NWAs Electrodes before and after 25,000 cycles (inset is the enlarged view of the Nyquist diagram and equivalent circuit).

In order to determine the changes about the resistance of the Co_3_O_4_ NWA electrode, the EIS analysis are applied before and after 25,000 cycles (Figure 6). The Nyquist plot is divided into the high-frequency region of the curve portion and the low-frequency region of the straight-line portion. The high-frequency region is controlled by the electrode reaction kinetics to represent the charge transfer resistance (R_ct_), and the low-frequency region can be stand for the diffusion resistance (W) between the electrolyte and the electrode material. In addition, the intersection point between the impedance curve and the horizontal axis is the intrinsic resistance (R_s_) of the electrode (Xiong et al., 2012; Yang et al., 2013; Cai et al., 2014; Xu et al., 2016). The inset of Figure 6 shows that the intrinsic resistance of Co_3_O_4_ NWAs decreases after 25,000 cycles (from 0.72 to 0.66 Ω). It may be that a portion of the Co_3_O_4_ NWAs breaks away from the Ni foam during the cycles, resulting in the intrinsic resistance decrease. The R_ct_ (from 1 to 1.66 Ω) and W (from 0.06 to 0.21 Ω) before and after the cycle were observed to increase. This phenomenon is probably due to the breakage of fewer nanowires during the cycles, leading to the destruction of the path with the smallest electrical resistance transported between the Co_3_O_4_ NWAs and Ni foam and thereby increasing the charge transfer resistance and diffusion resistance. In general, the impedances are approximately the same before and after 25,000 cycles, which further proves that the gully-network structure of Co_3_O_4_ NWAs possess excellent cycling stability.

**Figure 7:**
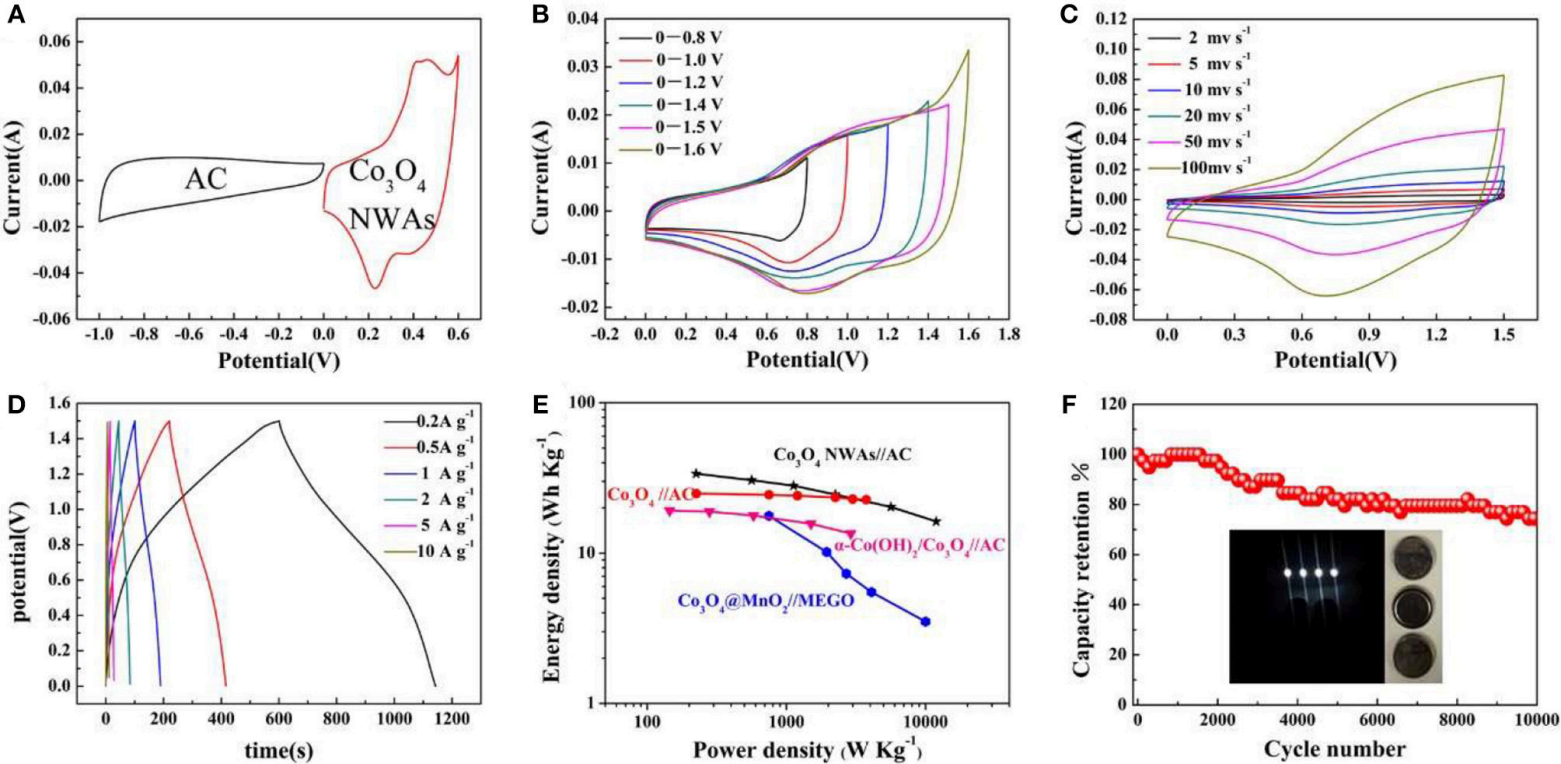
**(A)** The CV curve of AC electrode and Co_3_O_4_ NWAs electrode at a scan rate of 20 mV s^−1^, **(B)** CV curves of Co_3_O_4_ NWAs//AC device at different potential ranges, **(C)** CV curves of Co_3_O_4_ NWAs//AC device at different scan rates, **(D)** GCD curves of Co_3_O_4_ NWAs//AC device at different current densities, **(E)** Ragone plot of the Co_3_O_4_ NWAs//AC device, **(F)** Cycling performance of the Co_3_O_4_ NWAs//AC device at a current density of 2 A g^−1^ (inset is the photo of the lighted LED by the double Co_3_O_4_ NWAs//AC device).

To explore the practical application of Co_3_O_4_ NWAs electrodes, the Co_3_O_4_ NWAs//AC hybrid-supercapacitor based on the Co_3_O_4_ NWAs positive electrode and AC negative electrode is successfully assembled. Figure 7A shows the CV curves of the Co_3_O_4_ and AC electrode materials at a scan rate of 20 mv s^−1^ in a three-electrode system. The voltage window of the AC negative electrode ranges from −1 to 0 V, and the curve exhibits a typical double-layer behavior with a quasi-rectangular shape. Meanwhile, the positive voltage window of the Co_3_O_4_ NWAs electrode is 0–0.6 V, and the obvious redox peaks suggest the faraday behavior. The CV curves of the voltage window ranging from 0–0.8 V to 0–1.6 V for the Co_3_O_4_ NWAs//AC hybrid-supercapacitor device at a scan rate of 20 mv s^−1^ are shown in Figure 7B. The area of the CV curve becomes larger as the voltage window increases and no obvious redox peaks appear, indicating a good capacitive behavior. A significant polarization occurs when the voltage window increases to 1.6 V, so the voltage window choose to be 0–1.5 V. Figure 7C displays the CV curves at different scan rates under 0–1.5 V, presenting that a square-like curve remains similar at high scan rates. The GCD curves at different current densities are shown in Figure 7D, which displays good supercapacitive behavior and agrees with the CV curve results. A specific capacity of 162 C g^−1^ at 0.2 A g^−1^ of the device can be achieved and still remains 78 C g^−1^ at a high current density of 10 A g^−1^. Figure 7E is Ragone plots of the Co_3_O_4_ NWAs//AC devices based on the GCD discharge time, which shows that an energy density of 33.8 W h kg^−1^ at a power density of 224 W kg^−1^ is obtained. Even at a high power density of 12,000 W kg^−1^, an energy density of 16.25 W h kg^−1^ can also be kept. The energy and power densities of the Co_3_O_4_ NWAs//AC hybrid-supercapacitor are much higher than those of some other electrode materials, such as Co_3_O_4_//AC (24.9 Wh Kg^−1^ at 225 W Kg^−1^) (Zhang et al., 2013), α-Co(OH)_2_/Co_3_O_4_//AC (19.2 Wh Kg^−1^ at 145 W Kg^−1^) (Jing et al., 2014), Co_3_O_4_@MnO_2_//MEGO (17.7 Wh Kg^−1^ at 750 W Kg^−1^) (Huang et al., 2014). To evaluate the practical application of the device, the cycle performance of the hybrid-supercapacitor device is measured at a current density of 2 A g^−1^ (Figure 7F). It can be observed that the hybrid-supercapacitor devices assembled with Co_3_O_4_ NWAs also exhibits excellent cycle behaviors, maintaining a specific capacity of 74% after 10,000 cycles. The gully-network structure of Co_3_O_4_ NWAs materials ensures the stability of the structure and reduces the loss of capacity during the cycles. Moreover, the Co_3_O_4_ NWAs//AC hybrid-supercapacitor devices successfully light four LED lamps (inset in Figure 7F), illustrating that the as-prepared Co_3_O_4_ NWAs//AC devices can be wildly utilized for practical applications.

The admirable electrochemical behaviors of the Ni foam supported Co_3_O_4_ battery-type electrode materials for hybrid-supercapacitor can be ascribed to the following aspect: Firstly, the highly stable gully-network of Co_3_O_4_ NWAs effectively alleviates the volume expansion and contraction during the long cycle measurements and prevents the agglomeration of active materials, generating the outstanding cycle stability. Meanwhile, its porous structure enables the electroactive material to be completely exposed in the electrolyte solution, which facilitates the diffusion of ions and electrons, and allows strong storage of ions, resulting in excellent cycle stability. Secondly, the highly porous interconnected nanowire arrays consisted of numerous highly crystalline nanoparticles can greatly facilitate electrolyte transport and increase the surface area, thus promoting the electrolyte to easily diffuse into the inner region of the electrode. Finally, the direct growth of Co_3_O_4_ NWAs on Ni foam can significantly improve electrical conductivity, creating an expressway for electric charge transfer and resulting in desirable rate capability.

## Conclusion

3D gully-network structure Co_3_O_4_ NWAs directly grown on Ni foam have been successfully prepared via a facile and scalable hydrothermal procedure followed by calcination treatment. The unique porous gully-network structure of the Co_3_O_4_ NWAs electrode can offer large surface area, provide open channels for efficient ion transport as well as adaptability to the volume expansion and contraction during electrochemical reactions, which greatly improve the specific capacity, rate capability, and cycling stability of the Co_3_O_4_ NWAs electrode. When evaluated as a binder-free electrode for battery-type supercapacitor, unexceptionable faraday behaviors in terms of high specific capacity (582.8 C g^−1^ at a current density of 1 A g^−1^), desirable rate capability (with capacity retention about 84.8% at 20 A g^−1^), and outstanding cycle performance (93.1% capacity retention after 25,000 cycles) can be achieved. More remarkably, an energy density of 33.8 W h kg^−1^ at a power density of 224 W kg^−1^ and wonderful cycling stability with 74% capacity retention after 10,000 cycles can be acquired based on the hybrid-supercapacitor with the as-prepared Co_3_O_4_ NWAs positive electrode. All the excellent supercapacitive behaviors illustrates that our unique gully-network structure Co_3_O_4_ nanowire arrays hold a great promise for constructing high-performance energy storage devices.

## Author Contributions

CG design and performed the experiments. CG, MY, CW, JL, and CS prepared the samples and analyzed the data. CG, CJ, TL, LH, and YW participated in interpreting and analyzing the data. All authors read and approved the final manuscript.

### Conflict of Interest Statement

The authors declare that the research was conducted in the absence of any commercial or financial relationships that could be construed as a potential conflict of interest.
