# Supplementary material for: Highly Stable Gully-Network Co3O4 Nanowire Arrays as Battery-Type Electrode for Outstanding Supercapacitor Performance
Source: Front Chem. 2018 Dec 21;6:636. doi: 10.3389/fchem.2018.00636 (PMC6308209; doi:10.3389/fchem.2018.00636)
Supplement: Supplementary file 1 [file Presentation_1.pdf]

Highly Stable Gully-network Structure Co_3O_4 Nanowire Arrays as Battery-type Electrode for Outstanding Supercapacitor Performance

Chunli Guo[1*], Minshuai Yin[1], Chun Wu[2], Jie Li[1], Changhui Sun[3], Chuankun Jia[2,4*], Taotao Li[1], Lifeng Hou[1], Yinghui Wei[1,5],

[1.]College of Materials Science and Engineering, Taiyuan University of Technology, Taiyuan, Shanxi, 030024, P.R. China

[2]College of Materials Science and Engineering, Changsha University of Science & Technology, Changsha, 410114, China

[3]School of Chemistry and Chemical Engineering, Qilu Normal University, Jinan, 250200, P.R. China

[4]Key Laboratory of Advanced Energy Materials Chemistry (Ministry of Education), Nankai University, Tianjin, 300071, China

[5]College of Materials Science and Engineering, Taiyuan University of Science and Technology, Taiyuan, Shanxi, 030024, P.R. China

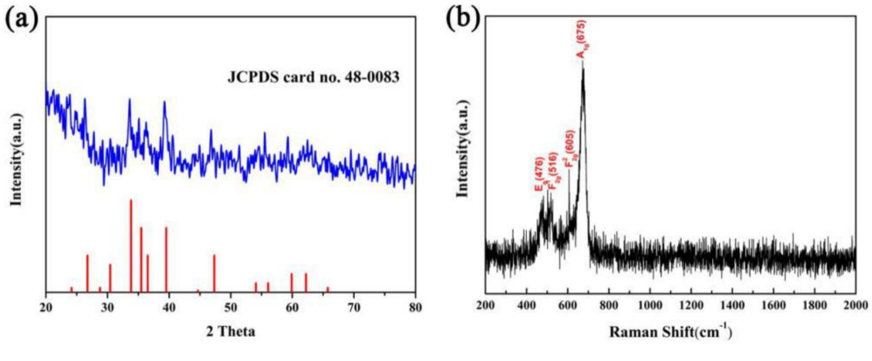

Figure S1. (a) XRD patterns of precursor powders, (b) Raman spectra of Co_3O_4 NWAs.

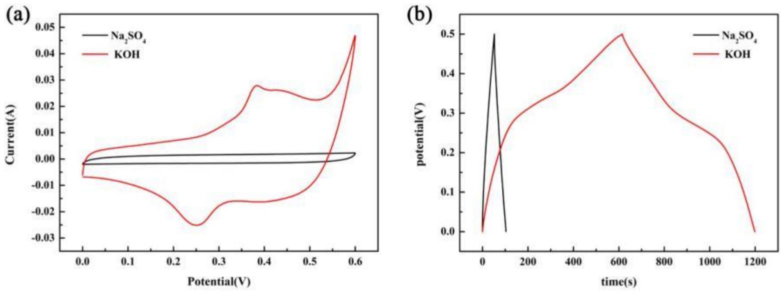

Figure S2 CV (a) and GCD (b) curves of Co_3O_4 NWAs electrodes in 6M KOH and 0.5M Na_2SO_4 at a scan rate of 20 mV s^{-1} and at a current density of 1 A g^{-1}, respectively.
